# Ten years of nurse-initiated antiretroviral treatment in South Africa: A narrative review of enablers and barriers

**DOI:** 10.4102/sajhivmed.v22i1.1196

**Published:** 2021-03-11

**Authors:** Talitha Crowley, Elizabeth Mokoka, Nelouise Geyer

**Affiliations:** 1 Department of Nursing and Midwifery, Faculty of Medicine and Health Sciences, Stellenbosch University, Cape Town, South Africa; 2 Forum of University Nursing Deans of South Africa (FUNDISA), Pretoria, South Africa; 3 Nursing Education Association, Pretoria, South Africa; 4 Department of Nursing Education, University of the Witwatersrand, Johannesburg, South Africa

**Keywords:** nurse-initiated, NIMART, South Africa, antiretroviral treatment, enablers, barriers

## Abstract

**Background:**

The roll out of nurse-initiated and managed antiretroviral treatment (NIMART) was implemented in 2010 by the National Department of Health (NDoH) in South Africa in response to the large numbers of persons living with HIV who needed treatment. To enable access to treatment requires shifting the task from doctors to nurses, which had its own challenges, barriers and enablers.

**Objectives:**

The aim of this narrative is to review content on the implementation of NIMART in South Africa over the period 2010–2020, with a focus on enablers and barriers to the implementation.

**Method:**

A comprehensive search of databases, namely, PubMed, Google Scholar and Cumulative Index to Nursing and Allied Health Literature (CINAHL), yielded qualitative, quantitative and mixed-method studies that addressed various topics on NIMART. Inclusion and exclusion criteria were set and 38 publications met the inclusion criteria for the review.

**Results:**

Training, mentorship, tailored tuberculosis (TB) and HIV guidelines, integration of services and monitoring and support have enabled the implementation of NIMART. This resulted in increased knowledge and confidence of nurses to initiate patients on antiretroviral treatment (ART) and decreased time to initiation and loads on referral facilities. Barriers such as non-standardised training, inadequate mentoring, human resource constraints, health system challenges, lack of support and empowerment, and challenges with legislation, policy and guidelines still hinder NIMART implementation.

**Conclusion:**

Identifying barriers and enablers will assist policymakers in implementing a structured programme for NIMART in South Africa and improve access, as well as the training and mentoring of professional nurses, which will enhance their competence and confidence.

## Introduction and background

The implementation of nurse-initiated and managed antiretroviral treatment (NIMART) was a direct response to the high rate of persons living with HIV and requiring treatment. Initially, antiretroviral treatment (ART) was provided in hospitals and initiation was performed by doctors. With more patients requiring treatment, as HIV infections soared and doctors’ capacity exceeded, a task shifting model was implemented, with nurses in the public sector having to initiate ART to scale up HIV treatment and increase access for more South Africans living with HIV.^1^ Poor socio-economic conditions and distances that patients had to travel to access care brought the need to decentralise HIV management services to primary healthcare (PHC) facilities. This, in turn, increased pressure to have more nurses trained to initiate ART and manage stable patients following national guidelines.^2^

The evidence that task-shifting may improve health outcomes, quality of care and patient satisfaction,^3,4^ together with the additional benefits of decentralisation of treatment^5^ and the growing numbers of persons living with HIV in South Africa, necessitated the wide-scale implementation of NIMART training. The World Health Organization recommendations and guidelines for task-shifting^6^ advocate that task-shifting should be implemented alongside efforts to increase the skilled workforce, health systems reorganisation and an enabling regulatory framework. Continued quality of care can only be maintained with standardised competency-based training, supportive mentoring and effective referral systems.^6^ Whilst there has been evidence that NIMART-trained nurses can initiate and manage patients successfully, researchers cautioned that we may not know enough about key patient-, provider- and organisational-level enablers and barriers of wide-scale implementation.^7^

It has been 10 years since the initial NIMART implementation in South Africa in 2010. Human immunodeficiency virus treatment and management guidelines have been revised several times since the ART implementation, and HIV care has been integrated into various other services such as general PHC,^8^ tuberculosis (TB) management^9^ and antenatal care.^10^ Nurse-initiated and managed ART has also expanded to include the management of children and patients with virological failure.^11^

With such wide-scale implementation and the evolving role of nurses in the context of NIMART and HIV management, it is inevitable that there may be challenges. Recent reviews that have summarised the enablers and barriers of the implementation of NIMART in the context of South Africa could not be found in the literature. It therefore became critical to search the literature in order to identify enablers and barriers and to make recommendations that will improve NIMART implementation.

## Aim

The aim of the article is to review published literature on NIMART in South Africa, with particular focus on the enablers and barriers to implementation.

## Method

In order to provide a comprehensive synthesis of the evidence and a broad perspective on NIMART, articles on the topic, its history and development were searched and presented in a narrative format.^12^ A narrative overview or review is a non-systematic narrative synthesis of previously published literature.^12^

### Search strategy and study selection

PubMed, CINAHL and Google Scholar databases were searched for relevant South African articles published between January 2010 and June 2020. Different search strategies were applied, using the MESH term combinations. In PubMed and CINAHL, we used Boolean operations such as ([‘nurse-initiated’] AND NIMART) AND ‘South Africa’) and in Google Scholar we used a string (NIMART South Africa). We identified additional records by reviewing master’s or PhD e-theses, conference abstracts and published studies known to the authors. Relevant grey literature, such as Department of Health documents, was also included.

One of the authors and a research assistant screened the abstracts for relevancy. Articles were included if they met the following criteria: published in English, between January 2010 and June 2020 and reported studies conducted in South Africa. Records were excluded if the results did not relate directly to NIMART or if the study was not conducted in South Africa.

### Data extraction and evidence appraisal

Data on the study aim, methods, sample and key enablers and barriers were extracted in tabular format. The quality of evidence was appraised using the John’s Hopkins Evidence Level and Quality Guide.^13^

### Ethical consideration

This article followed all ethical standards for research without direct contact with human or animal subjects.

## Results

A total of 479 records were identified: 7 from PubMed and CINAHL, 475 from Google Scholar and 15 through the authors of this article. After removing duplicates and excluding studies not relevant to the topic, 38 publications were included in our narrative literature review. The review includes qualitative, quantitative and mixed-methods studies and literature reviews that reported on evidence related to the NIMART implementation in South Africa.

Almost all the studies were classified as level III, B (non-experimental studies of good quality).^13^ Most of the quantitative and qualitative studies were descriptive in nature. However, the studies had sufficient sample sizes and provided reasonably consistent results and recommendations.

The studies’ findings are presented narratively under the headings ‘enablers’ and ‘barriers’ to NIMART implementation. A summary of the included studies is provided in Annexure 1. A summary of the enablers and barriers is depicted in Table 1.

**TABLE 1:** Summary of enablers and barriers to the implementation of nurse-initiated and managed antiretroviral treatment in South Africa.

Enablers and barriers	Key themes
**Enablers**	
Training and mentorship	Adequate training and mentorship results in increased knowledge, confidence and empowerment of nurses Training results in increased ART initiations and decreased load on referral facilities Patients are satisfied and retention in care is improved
HIV and TB management guidelines	User-friendly, easy to follow guidelines may contribute to improvements in HIV and TB care Continuous training on guidelines promote the appropriate use thereof
Integration of services	Integration of NIMART in primary, antenatal and TB care results in increased ART initiations and reduced time to initiation
Monitoring and support	Feedback about performance and teamwork amongst nurses promotes NIMART implementation
**Barriers**	
Non-standardised training and inadequate mentoring	Training programmes that are not standardised and the lack of continuous mentoring and professional development, leads to knowledge and confidence gaps amongst NIMART-trained nurses
Human resource constraints	Integration of NIMART with other services results in increased workloads, administrative duties and the performance of non-nursing tasks There is a lack of further delegation of tasks to lower cadres and the implementation of alternative models of care
Health system challenges	More services are provided whilst there are still a lack of infrastructure, resources, referral systems, data management and quality improvement systems
Lack of support and empowerment	Ineffectual managerial and doctor support as well as negative attitudes from colleagues and patients results in disempowerment
Challenges with legislation, policy and guidelines	There is no legislative framework or formal scope of practice for NIMART-trained nurses Guidelines are not always updated and adhered to
Patient-related factors	Poverty, stigma, lack of transport and non-adherence to treatment compromise NIMART outcomes

ART, antiretroviral treatment; HIV, human immunodeficiency virus; TB, tuberculosis; NIMART, nurse-initiated and managed antiretroviral treatment.

### Enablers to nurse-initiated and managed antiretroviral treatment implementation

The Streamlining Tasks and Roles to Expand Treatment and Care for HIV (STRETCH) trial, conducted before the official implementation of NIMART in 2010, reported on key enablers in the South African setting.^14^ Key components of effective implementation included tailored guidelines, training and support to build the clinical confidence of nurses and health services reorganisation.^14^ Since then, several other studies have highlighted similar enablers. We identified key enablers of NIMART implementation as being: (1) training and mentorship; (2) HIV and TB management guidelines; (3) integration of services; and (4) monitoring and support.

### Training and mentorship

In order to enable NIMART implementation, nurses need additional training. Nurses undergoing NIMART training have to complete HIV training covering various topics, clinical guideline training (Practical Approach to Lung Health and HIV/AIDS in SA [PALSA PLUS] and Integrated Management of Childhood Illnesses [IMCI]), and complete a portfolio of evidence (POE) containing a range of competencies. Nurse-initiated and managed antiretroviral treatment nurses are required to initiate and follow up a minimum number of patients in various age groups, including adults and children.^15^ Only after the successful completion of the POE, nurses receive a certificate of competence to initiate NIMART. In some settings, nurses’ competence are formally tested through objective structured clinical examinations (OSCE).^15^

With the introduction of the Department of Health’s Clinical Mentoring Manual for Integrated Services,^16^ mentorship was formally introduced to enhance clinical expertise. The model advocates a Clinical Proficiency Pathway starting with didactic training accompanied by clinical practice, assessment and continuous mentoring to ensure clinical expertise. Doctors or nurses can become clinical mentors, provided that they undergo mentoring training.^17^

Many non-governmental organisations (NGOs) and other private organisations rose to the task of assisting the National Department of Health (NDoH) with training and mentoring. These courses included, for example, the Clinical Competency in Antiretroviral and Tuberculosis (CCART) course developed by the University of Stellenbosch in collaboration with John’s Hopkins University and the United States Agency for International Development (USAID),^18^ a course presented by the Foundation of Professional Development (FPD)^19^ and a course developed by the University of KwaZulu-Natal.^2^ Across the courses, various challenges were reported such as difficulty to complete assignments, dispensing certificates and POEs for ART initiations.^18^ Success stories included improvement in pre-and post-course knowledge and confidence and increased rates of POE completion because of a team of roving mentors.^2,18,19^

Adequate training results in improved knowledge of HIV management, greater confidence and clinical competence, particularly if accompanied by mentoring.^14,20,21,22^ A study conducted in an urban and rural district in the Western Cape found that the majority of NIMART-trained nurses had adequate HIV management knowledge and were very confident to manage adult patients.^22^

The survey was specific to adult HIV management as nurses are only involved to a limited extent in the management of children in the Western Cape.

Nurse-initiated and managed antiretroviral treatment training leads to feelings of empowerment because of expanded roles.^20^ Appropriate training and support can lead to increased quality of patient care, confidence and professional development.^17^ Nurses may experience work satisfaction because of the difference they are making in patients’ lives.^23^ In the North West province, the ability to work independently boosted nurses’ self-esteem and self-worth.^24^ In Venda, some nurses reported that they felt proud that they were contributing to the NIMART programme.^25^

In Johannesburg, the training of nurses in NIMART increased access to ART as shown by an increase in the number of monthly initiations. It also resulted in reduced workloads at referral facilities.^26^ A qualitative study in Johannesburg reported that referrals to tertiary hospitals were significantly reduced after the introduction of NIMART. Nurses observed an improvement in the quality of life of their patients and the retention of patients in care, which they felt reflected the success of NIMART.^27^ Similarly, in KwaZulu-Natal, a cross-sectional study revealed that in 98% of the primary care clinics in one district, nurses initiated patients on ART. The majority of nurses also indicated that children were initiated in their clinics, with only some still being initiated by a doctor.^28^ Accelerating NIMART in paediatric patients, coupled with mentoring and support were considered enablers in the provision of ART in infants and children living with HIV in the Eastern Cape.^29^ Clinical exposure further contributes to knowledge and confidence as was found in a study conducted in the Western Cape where a higher caseload of HIV patients was associated with higher knowledge and confidence.^22^ In the North West province, effective placement, specifically at Community Health Centres, contributed to POE completion.^24^

The mentoring of nurses had a positive effect on patient care by improving the clinical skills of professional nurses and by raising the standard of HIV care in the Eastern Cape.^30^ In Khayelitsha, Cape Town, a mentoring programme led to improvements in the quality of care nurses provided and their knowledge and confidence.^17^ Nurses initiated 77% of ART-eligible patients after completing a mentorship programme and improvements in their ART management were observed.^17^ If the majority of persons living with HIV are managed by nurses, doctors can attend to the more complicated cases, which, in turn, may result in better overall patient outcomes. Another study in the Western Cape found that a 2-week mentoring period was associated with greater HIV confidence compared with other periods. A period of 2 weeks of ‘dedicated mentoring-time’ with a mentor may be sufficient to complete competencies.^22^

Jobson et al.^31^ conducted a qualitative study, evaluating the role of mentoring in the support of the scale-up of ART across three provinces of South Africa. The study explored the role of targeted mentoring using a needs-based approach to support NIMART, pharmacy management and data management.^31^ The study adopted a two-stage approach, which they classified as proactive and reactive mentoring. For NIMART-trained nurses, the proactive approach entailed having nurse mentors accompanying them during patient consultations, which provided support in transferring skills learnt during training to actual implementation when providing care. Participants stated that they had gained knowledge after training and mentoring, which empowered them to up-scale services through NIMART. The reactive role required the mentor (nurse) to be a problem solver and a source of support, as required at various stages during HIV care and management. This type of support is important for NIMART-trained nurses who were starting to initiate and manage patients on ART. Mentorship strengthened skills and knowledge transfer, allowed mentees to develop their own skills and provided a source for psychosocial support as mentees became confident and felt empowered to take on new responsibilities in providing HIV care.^31^

### HIV and TB management guidelines

The introduction of tailored guidelines such as the Practical Approach to Care Kit (PACK) was critical to assist nurses to implement NIMART.^14^ A mixed-methods study conducted in the Limpopo province found that nurses practiced rational prescription and followed ART guidelines, with lower patient mortality (below 1%) and loss-to-follow-up rates compared with that of surrounding hospitals. In addition, 91.1% of NIMART-managed patients had undetectable viral loads after 1 year on treatment. Patients also reported high levels of satisfaction.^32^ A study conducted in the North West province found that nurses preferred guidelines to be user-friendly, easy to follow and keep on their person and available in all consulting rooms.^33^ Nurses also wanted to be supported and supervised until they are familiar with applying the guidelines in practice. They preferred continuous training and education about guidelines. Organisational and structural changes such as manageable workloads and improved communication were thought to enable adherence to ART and TB treatment guidelines.^33^

### Integration of services

Initially, NIMART was implemented as a vertical programme. Now it is increasingly being integrated into a range of primary care services.^8^ The integration of HIV care into PHC services is reported as a structural facilitative factor for the implementation of NIMART.^27^

The integration of HIV care into antenatal care and the introduction of option B+ meant that all pregnant women living with HIV needed to be initiated on ART. A retrospective record analysis conducted in the Gauteng province comparing time to initiation of ART amongst antenatal clients before and after NIMART implementation showed no significant reduction in the time to initiation on ART.^10^ However, the study was conducted in the early stages of NIMART training and not all midwives may have been trained; therefore, they could only initiate clients on certain days.

Contrary to this, a study conducted in KwaZulu-Natal, soon after NIMART implementation in 2011, found that 97% of women were initiated on ART in antenatal care settings, illustrating a shift in care from ART clinics to nurse-managed antenatal clinics. This also resulted in reduced time to ART initiation from 38 to 4 days.^34^ One study evaluated the effectiveness of the NIMART programme in the Waterberg district of the Limpopo province and found the number of patients initiated in the hospital dropped as did the number lost-to-follow-up, after the introduction of NIMART in primary care.^35^

### Monitoring and support

A study conducted in the Western Cape found that regular feedback about clinic and personal performance was associated with higher HIV management knowledge,^22^ with a study in Limpopo highlighting that support from visiting doctors and management were viewed as very helpful, even if management could not resolve all their problems.^23^ Support amongst nurses is a further enabler to the implementation of NIMART.^25^ Supportive teamwork (ongoing support from facility managers and colleagues), motivation and support from mentors were found to be key contributors to POE completion in the North West province.^24^

### Barriers to nurse-initiated and managed antiretroviral treatment implementation

The implementation of NIMART may have been too hasty, not providing enough time for crucial capacity-building interventions such as mentoring and systems reorganisation.^20^ Barriers identified in this review include: (1) non-standardised training and inadequate mentoring, (2) human resources constraints, (3) health system challenges, (4) lack of support and empowerment, (5) challenges with legislation, policy and guidelines and (6) patient-related factors.

### Non-standardised training and inadequate mentoring

Inadequate or non-standardised *training* and the lack of continuous *clinical mentoring* and supervision by clinic managers were mentioned in most studies as barriers to the implementation of NIMART.^19,20,23,24,25,27,28,35,36,37^

As mentioned before, NIMART training was, and is being conducted by various NGOs, the NDoH and private training providers. One of the challenges is that *training is not standardised*, ranging from a few days to a few weeks or even months, which makes it difficult to evaluate its effectiveness or quality. Each provider determined their own programme’s content, duration, instruction methods and assessment strategies. This limits the ability to set guidelines for structured training and mentoring. In some cases, nurses initiated patients even before attending or successfully completing NIMART training.^19^ The process of submitting POE’s and doing an additional course in dispensing was found to be dysfunctional as many nurses could not complete it.^15,18,38^ In the North West, prerequisites such as PACK training and IMCI were barriers to the completion of POEs.^24^ In rural North West province, challenges relating to NIMART training included the lack of a standardised curriculum, lack of involvement of quality assurance bodies, nursing colleges and universities and inadequate continuous professional development (CPD).^39^ Another barrier identified in the North West province was disorganisation at the level of the Regional Training Centre (not receiving POE’s directly after the training) and at the level of the trainee (lack of planning).^24^ This partly defeated the aims of NIMART, which was meant to be an intervention intended to improve healthcare access and equity, ideally without compromising the quality of care and a key strategy for expanding access to HIV treatment at PHC level.

There is a lack of evidence in South Africa regarding the effectiveness of different NIMART training programmes and the impact on patient outcomes.^2^ One should also question the sufficiency of a once-off NIMART training and further explore the role of continuing education and CPD. A study conducted in the Western Cape found that NIMART nurses with recent training on guidelines (less than 3 years) had better knowledge compared with nurses who reported to be trained more than 3 years ago.^22^ In general, NIMART-trained nurses identified the need for continuing education.^2,19,20,21,35,39^

Whilst NIMART training enabled access, a *lack of mentoring* following training was a barrier in terms of the gap between the number of nurses who received training and those who could initiate treatment. Despite the large-scale training to upskill nurses, not all nurses initiated ART after receiving training. A study conducted in rural North West province found that although NIMART increased access to care, there was no steady increase in the initiation of adults, children and pregnant women on ART. Initiation was especially low amongst children. This was despite the fact that 75% of nurses in 99% of healthcare facilities were NIMART trained.^40^ Facilities were also performing below the targets for retention in care and viral load completion and suppression rates, indicating poor quality of care and non-compliance to guidelines. Another study conducted amongst NIMART-trained nurses across 7 provinces found that of the 126 nurses sampled, only 79 initiated treatment and only 9 initiated treatment in children.^19^

Factors contributing to low initiation rates include inadequate training, lack of confidence and lack of mentoring and supervision. A study conducted in the Western Cape amongst 77 NIMART nurses working across 29 healthcare facilities on factors influencing the knowledge and confidence of professional nurses prescribing HIV treatment concluded that training, mentorship and clinical practice experience were associated with confidence and knowledge.^22^ A qualitative study conducted in Johannesburg found that a lack of mentoring was likely to have contributed to a lack of nurses’ confidence to initiate ART. They recommended a nurse-mentor model where experienced nurses can supervise and support colleagues; as well as access to telephonic support.^20^ Nurse-initiated and managed antiretroviral treatment-trained nurses in Venda reported that because of the limited 1-week didactic training, they ended up learning most of the competencies at work, highlighting the need for mentoring support.^25^ The need for supportive mentoring was underscored by the fact that only 63% of the nurses who completed a training course in KwaZulu-Natal felt confident enough to initiate ART following the training.^2^ With the escalating number of persons living with HIV requiring treatment, it became crucial to nationally upskill the nurses who were not initiating as a result of a lack of mentorship and standardised training.

Whilst some of the NGOs had a mentorship programme as part of their training, this was not a norm across all trainings. The NDoH subsequently developed a national Clinical Mentorship Programme, which was aimed at providing practical on-site support to NIMART-trained nurses and ensure a competent and confident workforce. A manual was developed, with didactic content and practical tools that could be used to design, implement and evaluate a clinical mentorship programme at facility level and following clinical guidelines on HIV care. Of importance was that the manual could also be used by pharmacists, medical officers and other members of the multidisciplinary team providing HIV care services.^16^ However, even with this Clinical Mentorship Programme in place, challenges with regard to mentoring exist. One of these challenges include that during proactive mentoring, it often happened that the mentors ended up delivering the actual service themselves and were sometimes not available when needed because of being responsible for several facilities.^31^ There is also no standardised time or method for clinical mentorship and it varies from a minimum of 40 h in the Western Cape^17^ to targeted mentoring in other settings.^15,31^

Several studies identified key *knowledge and confidence gaps* amongst NIMART-trained nurses, further indicating a need for improved training and mentoring. A lack of confidence to manage children was identified in a study in the North West province.^39^ Naude^21^ similarly found low levels of competency in certain areas of HIV management, particularly for ART initiation and follow-up in children, in the North West province. In Venda and Limpopo, nurses reported that they faced problems in performing certain tasks such as obtaining blood from children.^25,41^

Mashudu^35^ also found that few children were initiated on ART in Limpopo. Low initiation rates amongst children should be investigated further. It may be that the policy in many provinces, such as the Western Cape, is that children must be managed by doctors or that paediatric NIMART training and mentoring is not sufficient. In a study to explore the experiences of healthcare professionals regarding the provision of ART for children in PHC settings in Nelson Mandela Bay Health District in the Eastern Cape, the need for training, mentoring and debriefing was expressed as one of the challenges related to providing decentralised ART to children. Nurses were apprehensive to work with children and noticed incongruence in the interpretation of ART side effects in children.^29^

An analysis of nurses’ queries to the National HIV and TB Health Worker Hotline showed that 66% of the queries received were from NIMART-trained nurses and most were related to ART initiation and adverse drug reactions. The most common knowledge gap identified was on the interpretation of blood results before initiation of ART,^42^ which was also confirmed by Rasalanavho.^25^ In the study conducted in the Western Cape, low confidence was reported in prescribing for concurrent illnesses and in identifying the signs and symptoms of immune reconstitution inflammatory syndrome. Nurses had less than optimal knowledge of virological failure and drug–drug interactions.^22^ In KwaZulu-Natal, most NIMART nurses knew the correct ART regimens, ART eligibility criteria and when blood for CD4 count and viral load should be taken, although worrying gaps were identified.^28^

### Human resources constraints

*Human resources* constraints, increased workloads and administrative duties such as paperwork, were cited in many studies as negatively influencing the implementation of NIMART,^14,19,20,21,23,25,27,32,35,39^ with doctors reportedly not fully supporting the NIMART programme.^25^

Excessive workloads also interfere with the completion of mentorship programmes.^24^

In one study, 55% of nurses reported seeing more than 30 patients a day and 30% saw more than 40 patients a day.^21^ As a result of integration of HIV management in primary care, NIMART-trained nurses also have other tasks to perform.^22,25^ Nurses reported the inability to delegate tasks to lower cadres and also reported having to do non-nursing tasks such as collection of drugs from depots^27^ or using their own transport to collect drugs.^25^

Despite human resource challenges, NIMART nurses display resilience. In the Western Cape, even though 44.4% of nurses felt that their workload was unacceptable, and 48.1% were dissatisfied with their work environment, salary and work hours; 88.3% were nonetheless still motivated to work.^22^ The challenge of high workloads was mitigated by nurses in Venda through problem-solving and innovative strategies such as allocating different times for collecting tablets and reviews as well as group counselling.^25^

The growing number of patients on ART necessitates looking at decentralising ART care further and utilising chronic care models for stable patients on ART to decongest clinics.^20^ Several studies mentioned the utilisation of lower cadres of healthcare workers such as lay workers to trace patients,^27^ the education of the community, increased community management of HIV and addressing poverty and stigma, as crucial to the continued success of the HIV programme in South Africa.^23,43^

### Health systems challenges

Although integration of services can be an enabling factor, it is not without challenges. High HIV–TB co-infection rates and high antenatal HIV prevalence rates in South Africa necessitate integration of HIV into TB and antenatal care. This means that primary care nurses and midwives have to expand their competencies to include NIMART. These changes also have an effect on the scope of practice and the training of nurses. It may also impact the quality of care that can be provided to other patient populations in primary care and women’s healthcare settings. A study conducted in the Free State to evaluate patients’ perceptions of the effect of the integration of NIMART into primary care on quality of care showed that there was no decrease in patient satisfaction with staff. This was despite increases in patient numbers. However, satisfaction scores were lower for child health and chronic care patients (except TB), suggesting that there may be a knock-on effect on other services.^44^

A study in the Limpopo province on the views of registered nurses of the prevention of mother-to-child transmission (PMTCT) programme revealed considerable challenges to integrate NIMART into antenatal and postnatal care.^43^ These challenges related particularly to having to spend more time with patients when initiating ART during the antenatal period; the additional workload was not accompanied by additional support.

Integration of services should be accompanied by the provision of adequate resources and reorganisation of services.^14^ However, several studies reported inadequate *infrastructure and resources* such as consultation rooms, inadequate workspace compromising patient privacy and confidentiality, as well as the lack of resources such as stationery, drugs, equipment needed for blood collection, telephones, electronic drug ordering systems, information systems and slow turnaround times of blood investigations.^15,19,21,23,25,27,32,33,35,41,43^ Poor data management, including lack of compliance with standard operating procedures, incomplete records and inadequate audits hampered performance.^23^ Nurses also experience poor referral feedback systems.^27^ There is a necessity for quality improvement teams to ensure quality of care.^20^

### Lack of support and empowerment

*Health system* issues and lack of *managerial support* was identified in several studies.^20,23,25,33^ In Venda, nurses reported a lack of support from doctors and refusal to see complicated cases requiring expert management.^25^

Naude,^21^ through a mixed-methods study, explored empowerment amongst NIMART-trained nurses. Quantitative findings revealed that nurses were only moderately structurally empowered.

With regard to psychological empowerment, professional nurses felt that they only minimally influenced their work environment. Management and organisational processes were identified as being central to the empowerment of NIMART nurses.^21^ In the Eastern Cape, barriers to paediatric NIMART implementation included ineffective management, disharmony and non-conducive work environments.^29^

A negative attitude towards the management of HIV and TB patients was identified by Mboweni and Makhado.^38^ Negative attitudes and discrimination from non-NIMART-trained nurses and refusal to manage HIV positive patients was also reported in a study conducted in Venda.

Nurses reported that patients questioned their ethical values to keep information confidential and had negative attitudes towards the NIMART programme.^25^ A study conducted in the Limpopo province found that nurses also had fears of infecting themselves with HIV,^41^ indicating that there may be a lack of workplace-based support.

### Challenges with legislation, policy and guidelines

The lack of enabling *legislation* and *regulations*,^45,46^ the lack of clear guidelines and standard operating procedures,^37^ unclear roles or scope of practice – nurses sometimes perform the roles of medical practitioners and pharmacists,^21^ and salary or remuneration challenges were also reported.^32^ Provincial governments and the NDoH were mandated to maintain records of all nurses authorised to prescribe ART and to communicate this to the South African Nursing Council. Anecdotal reports suggest that this has not happened. There is no formal scope of practice for NIMART-trained nurses and they do not receive additional remuneration for expanded roles.

Some studies reported the lack of updated guidelines, particularly in rural areas.^28^ A study in KwaZulu-Natal and North West provinces revealed that nurses may not be following HIV and TB guidelines. Barriers to adhering to guidelines included a lack of agreement with the guidelines or guidelines not being clear or understandable; insufficient knowledge as a result of not being updated or involved in guideline development; frequent guideline changes; poor motivation; and lack of supportive supervision.^9^ Continuous revision of guidelines requires frequent updated training, but training programmes are not always communicated timeously to ensure that staff can attend.^43^

### Patient-related factors

Many studies also highlighted patient-related factors hindering the implementation of NIMART such as poverty, stigma, lack of transport and non-adherence to treatment.^25,27^ However, the discussion of patient factors is beyond the scope of this review and could perhaps be a focus for future studies.

## Discussion

It has been 10 years since the implementation of NIMART in South Africa. Evidence from observational studies indicates that there are several enablers and barriers to implementation. Based on the evidence presented here, we discuss implications for practice, education and research.

### Implications for practice

The narrative review identified two frameworks developed from rigorous research that could be implemented in practice to strengthen NIMART training and implementation and empower NIMART-trained nurses.^21,37^ The conceptual framework for strengthening NIMART training and implementation developed by Mboweni and Makhado^39^ outlines structural attributes that advocates for adequate resources, infrastructure, supportive legislation and policy, effective training strategies and a supportive healthcare system culture.^37^ This is supported by the framework of Naude^21^ who outlined the following elements of structural empowerment: training and mentoring (knowledge, technical skills and professional growth); resources (human, equipment and supply chain); support (clinical supervision, guidance from managers and feedback); communication (information, protocols and guidelines) and power (formal in the form of role clarification and decision making and informal in the form of team acceptance and remuneration).^21^ Many of these infrastructural and resource inadequacies may be addressed by investing in and improving the leadership and management of PHC services.

### Implications for education

Mboweni and Makhado’s framework identified that effective NIMART education and implementation requires (1) an integrated curriculum and effective training strategies for in- and pre-service education and (2) a healthcare system culture that includes support through mentoring or coaching, discipline, communication and referral systems.^37^

Although NIMART training is still being provided by NGO’s and various private organisations, there is now a greater effort to incorporate comprehensive HIV training, including NIMART, in undergraduate pre-service programmes.^2,46,47,48^ Evidence suggests that pre-service nurses still have inadequate HIV management knowledge.^47^ Some researchers advocate for specialisation programmes for the management of chronic conditions, including HIV and TB.^21^ One study recommended future research regarding the role of the postgraduate diploma in Primary Care Nursing in the implementation of NIMART as nurses felt that those who had the qualification were better equipped to manage patients on ART.^27^ However, in the same study, as reported in other studies,^2,19^ the majority of NIMART nurses did not have a qualification in Primary Care (Health Assessment, Diagnosis, Treatment and Care). More research is needed to explore if a Primary Care qualification is important for NIMART implementation^2^ or whether NIMART competencies should be integrated in the undergraduate programme or the postgraduate diploma in Primary Care Nursing. Nonetheless, there is a need to standardise ‘in-service’ types of NIMART training across provinces, aligning them to the competencies outlined in the Clinical Mentorship Manual for Integrated Services,^16^ as well as ensuring formal competency assessment.

### Implications for research

Further research is needed on the standardisation and effectiveness of NIMART training and mentoring programmes and the long-term impact on patient outcomes towards attaining national and international targets for HIV care and management. Evidence-based guidelines for the integration of NIMART into primary care services and continuous monitoring and support are urgently needed.

### Strengths and limitations

The search strategy was not comprehensive and some relevant studies may have been excluded.

However, the authors are confident that we have provided an exhaustive description of barriers and facilitators to NIMART implementation in South Africa.

## Conclusion

Although training, mentorship, guidelines, integration of services and monitoring and support have enabled the implementation of NIMART, several barriers such as non-standardised training, inadequate mentoring, human resource constraints, health system challenges, lack of support and empowerment, and challenges with legislation, policy and guidelines still hinder its effectiveness.

Various key role players such as the NDoH, the South African Nursing Council, training providers and researchers need to work together to standardise training and provide evidence-based guidelines for mentoring, integration of services and continuous monitoring and support.

## Appendix

**ANNEXURE 1:** Summary of included studies.

Authors/year	Study aim	Methods	Sample	Key enablers and barriers	Level and quality of evidence
Cameron et al.^19^	To determine the percentage of nurses initiating new HIV positive patients on therapy within 2 months of attending the NIMART course and to identify possible barriers to nurse initiation.	Quantitative: Telephonic interviews, structured questionnaire	A total of 126 out of 1736 PHC nurses in 7 provinces	Enablers: training – 79 out of 126 initiating ART	Level III B
				Barriers: Low initiation in children (9 out of 126), staff shortages/workload, allocation of other tasks	
Colvin et al.^49^	To describe the expanding access to ART in South Africa and the role of nurse-initiated treatment	Opinion article pre-NIMART rollout summarising evidence from STRETCH trial	Literature	Enablers: Realignment of health system, systems strengthening	Level IV B
Crowley^18^ *(Conference proceedings)*	To determine the effect of a HIV/TB competency-based short course on professional nurses’ knowledge and confidence in managing patients with HIV and TB	Quantitative: pre- and post-test	A total of 65 students completed the pre-test and 56 completed the post-test	Enablers: Training and mentoring improving knowledge and confidence	Level III B
Davies et al.^20^	To explore nurse and facility and programme manager perceptions of NIMART implementation in Gauteng, South Africa	Qualitative: Interviews and focus groups	A total of 12 nurses and 18 managers involved in NIMART	Enablers: Nurse empowerment because of expanded roles	Level III B
				Barriers: Human resources, training, clinical mentoring, health systems issues	
Ford^45^ *(Master’s research report)*	To review and analyse the existing legal framework and provisions for NIMART in South Africa and to identify ethical issues and implications of NIMART within the current legal framework	A comparative analysis of literature	Literature	Enablers: NIMART framework founded by Constitution and enabled by health policy	Level IV B
				Barrier: Aspect of enabling legislation related to nurse training and accreditation required	
Georgeu et al.^14^	To develop a contextualised understanding of factors affecting the implementation of the NIMART programme (STRETCH trial)	Qualitative: Focus groups^16^ and interviews^25^	Nurses, patients, managers, coordinators, managers and site physicians	Enablers: Acceptability, confidence to deliver ART, support, training	Level III B
				Barriers: Changes in working and referral relationships, capacity and workload constraints, logistical and infrastructure challenges	
Green et al.^17^	To assess quality of care of clinical mentorship of NIMART in Khayelitsha, South Africa	Quantitative: A before-after cross-sectional study	Routine clinical data from 229 patient folders and 21 self-assessment questionnaires	Enablers: Mentoring, increased clinical confidence, professional development	Level III B
Hanrahan & Williams^43^	To determine what the registered nurses’ perspectives are on the PMTCT programme as implemented at four PHC facilities in the Limpopo province	Qualitative: Semi-structured interviews	A total of 21 nurses	Enablers: Education of staff, updates on the PMTCT programme guidelines and policies, effective communication with patients	Level III B
				Barriers: Increased workloads, staff shortages, poor planning of training, equipment and medication shortages	
Jobson et al.^31^	To understand the implementation process of targeted mentoring for clinical practice, data management and pharmacy management, at public healthcare facilities in South Africa	Qualitative: Structured interviews	A total of 74 healthcare workers from 3 South African provinces	Enablers: Mentoring improving self-efficacy, knowledge and skills transfer, psychosocial support	Level III B
				Barriers: Mentors responsible for several facilities, unavailable when help needed, over-dependence on mentors, lack of communication and planning, taking over clinical work	
Jones & Cameron^15^	To describe and analyse the achievements and evolution of clinical mentoring for NIMART-trained professional nurses by roving mentor teams in PHC facilities in the health districts of Tshwane (Gauteng province), Nkangala (Mpumalanga province) and Capricorn and Vhembe (Limpopo province) over a period of 5 years	Primarily qualitative: Semi-structured interviews. Data obtained from routine monitoring and evaluation reports, and from the DoH District Health Information System	A total of 92 professional nurses who had completed classroom training in NIMART, 20 facility managers, 4 subdistrict programme managers, 45 roving mentors and 12 Foundation of Professional Development (FPD) operational managers	Enablers: Targeted mentoring	Level III B
				Barriers: Low completion rates of training, large number of nurses requiring mentoring, lack of mentors/mentoring, lack of ongoing mentoring	
Jones et al.^30^	To evaluate the effect of a NIMART mentor	Quantitative: Record review	Existing pre-ART patient files (*n* = 286)	Enablers: Mentoring plays an important role in professional nurse training and support	Level III C
Lekhuleni et al.^47^	To determine the knowledge of student nurses in NIMART	Quantitative: Questionnaire	A total of 106 third and fourth level student nurses University of Limpopo	Barriers: Undergraduate training – students have insufficient knowledge on ensuring sufficient ART stock, TB screening on HIV positive patients and privacy during NIMART	Level III B
Mabelane et al.^23^	To identify the factors affecting the implementation of nurse-initiated ARV treatment in PHC clinics referring patients to Dr C.N. Phatudi Hospital, Limpopo province	Qualitative: Focus groups and interviews	A total of 15 registered nurses	Enablers: Support from management, visiting doctor, work satisfaction	Level III B
				Barriers: General lack of resources including healthcare workers, drugs, stationery, telephones, poor training, inadequate workspace	
Makhado et al.^33^	To determine factors facilitating trained NIMART nurses’ adherence to treatment guidelines: A vital matter in the management of TB/HIV treatment in South Africa	Qualitative: Focus groups	A total of 24 NIMART nurses	Enablers: Improved accessibility, usability (user-friendly) and availability of treatment guidelines, motivation, support and supervision, improved knowledge and awareness, organisational-structural changes	Level III B
Makhado et al.^9^	To explore and describe barriers to treatment guidelines adherence amongst nurses initiating and managing anti-retroviral therapy and anti-TB treatment in KwaZulu-Natal and North West provinces	Qualitative: Focus groups	A total of 24 NIMART nurses	Barriers: Lack of agreement with guidelines, poor motivation to implement, poor clinical support and supervision, insufficient knowledge or lack of awareness, organisational factors – time pressures, heavy workload, poor access to guidelines	Level III B
Mangi et al.^36^	To review and analyse literature on self-efficacy and clinical performance amongst professional nurses regarding quality of care in implementation of NIMART programme	Literature review	Literature	Barriers: Lack of mentoring, support and exposure to clinical practice had negative effect on nurses’ self-efficacy	Level III B
Mashudu^35^	To evaluate the effectiveness of the NIMART programme, Waterberg District, Limpopo province	Quantitative: Descriptive cross-sectional	All PHC clinics and NIMART nurses	Barriers: Workload, administrative duties, insufficient consultation rooms, human resources challenges, managerial support, mentoring, health system issues	Level III B
Mathibe et al.^8^	To explore clinicians’ perceptions and patients’ experiences of integration of antiretroviral treatment in PHC clinics	Mixed methods: Questionnaires and focus groups	A total of 4 PHC facilities; 35 clinicians; 4 focus groups with HIV positive patients	Enablers: Integration of care promotes access to care, prevention of stigma, staff development and support	Level III B
				Barriers: Workload, poor staff attitudes, poor communication	
Mboweni et al.^40^	To determine and evaluate the impact of NIMART training on HIV programme in order to make recommendations leading to effective training and implementation	Quantitative: Records analysis. (Ngaka Modiri Molema District, North West province)	The statistics of ART indicators were collected from the DHIS from January 2012 to December 2016	Enablers: NIMART training increase access	Level III B
				Barriers: Poor quality of HIV management, non-compliance to guidelines and monitoring of treatment effectiveness	
Mboweni & Makhado^37^	To develop a conceptual framework to strengthen NIMART training and implementation in the North West province to improve patients and HIV programme outcomes	Mixed methods: Explanatory, sequential	ART statistics from the DHIS & Tier.net of 10 PHC facilities and 5 focus group discussions amongst 28 NIMART nurses and 3 HIV programme managers	Barriers: Low ART initiation, poor monitoring on ART, human resource ratio’s, no framework to guide training and mentoring	Level III B
Mboweni & Makhado^39^	To explore and describe the challenges influencing NIMART training and implementation amongst professional nurses and programme managers. (Molema district, North West province)	Qualitative: Focus groups and individual interviews	A total of 28 NIMART nurses and managers directly involved in the programme	Barriers: Inadequacy of NIMART training, lack of a standardised curriculum, healthcare system challenges	Level III B
Mngqibisa et al.^2^	To evaluate the effectiveness of the NIMART course in increasing the knowledge of trainees in select clinical competencies	Quantitative: A single-group pre- and post-quasi-experimental design	A total of 369 trainees who had benefitted from the course during the implementation period	Enablers: Training improves knowledge in HIV and its management	Level III B
				Barriers: Need for on-the-job mentoring and support to maximise clinical outcomes	
Mnyani et al.^10^	To assess timing of antenatal care and ART initiation in HIV-infected pregnant women before and after introduction of NIMART	Quantitative: Records analysis	Records of 1436 ART-eligible pregnant women	Enablers: NIMART training decreases time to ART initiation	Level III B
Modeste & Adejumo^48^ *(Conference proceedings)*	Exploration of the integration of NIMART training in the pre-service nursing curriculum at one university in South Africa	Qualitative: Focus groups and individual interviews	A total of 52 nurse educations in 7 provinces in South Africa	Enablers: Training – educators have different views some preferring NIMART to be part of the postgraduate programme whilst others feel it should be done at pre-service level, current legal framework is enabling	Level III B
				Barriers: Limited knowledge related to pharmacology, ART, side-effects, interpretation of blood results amongst practicing nurses, gaining experience to provide NIMART upon graduation may be problematic	
Motlokoa^24^ *(Master’s thesis)*	To identify barriers and facilitating factors affecting the submission of POEs by NIMART-trained nurses in the North West province	Qualitative: Focus groups	A total of 30 NIMART Nurses in three focus groups	Enablers: Support, teamwork, effective placement and motivation	Level III B
				Barriers: Disorganisation, NIMART prerequisites, lack of human resources	
Mophosho^27^ *(Master’s thesis)*	To explore and describe perceptions of operational managers, facility managers and professional nurses on the facilitators and barriers to the implementation of NIMART in the City of Joburg (CoJ) clinics	Qualitative: Interviews	A total of 26 participants comprising operational managers, facility managers and professional nurses	Enablers: Adequate training – opportunities for continuing education, mentoring, NIMART guidelines, integration of NIMART into PHC services	Level III B
				Barriers: Shortages of health workforce, ART stock outs, poor referral feedback, food insecurity mobility of patients	
Naude^21^ *(PhD thesis)*	To develop a framework to empower professional nurses for NIMART therapy in PHC facilities in the North West province	Mixed methods: Questionnaires and interviews	A total of 182 professional nurses completed questionnaires and 20 interviews	Enablers: Knowledge and skills, mentoring and support, guidelines, positive psychological experiences, feedback from managers and evaluation of performance, conducive working environment, power to perform tasks	Level III B
				Barriers: Incompetence, training and clinical opportunities based on favouritism, negative psychological experiences, workload, lack of structural and psychological empowerment, clear role delineation needed, availability of drugs, equipment and consulting rooms	
Nozulu^34^	To evaluate ART initiation of pregnant women attending antenatal care in eThekwini district Community Health Centres (CHCs) between the Financial Years (FY) 10/11 (when NIMART was newly introduced) and FY13/14 (when NIMART was in full implementation)	Quantitative: Observational descriptive retrospective chart review	Records of pregnant women living with HIV that initiated ART	Enablers: A shift in point of care for ART initiation of pregnant women from ART clinics to nurse-managed antenatal clinic, reduced time to initiation	Level III B
Nyasulu et al.^26^	To determine if NIMART rollout to PHC facilities increases access to antiretrovirals in Johannesburg: An interrupted time series analysis	Quantitative: Interrupted time series analysis	A total of 20 535 ART-naïve patients from Region F of the CoJ who were initiated on ART from October 2009 to March 2012	Enablers: NIMART increase ART uptake and reduce workload on referral facilities, capacity building, training and mentoring	Level III B
Rasalanavho^25^ *(Master’s thesis)*	To explore and describe the challenges confronting professional nurses implementing the NIMART programme in PHC facilities under Thulamela B Municipality, Vhembe District	Qualitative: Interviews	A total of 15 professional nurses	Enablers: Health systems reorganisation, for example, appointment systems, club systems, peer support between nurses	Level III B
				Barriers: Shortage of infrastructure, medication, lack of support from management and non-NIMART-trained nurses, training (lack of skills to work with children), doctors not fully supporting the NIMART programme	
Rawat et al.^44^	To explore patient responses on quality of care and satisfaction with staff after integrated HIV care in South African PHC clinics	Quantitative: Surveys	A total of 910 patients and caregivers at two time points after integration in four clinics in Free State, South Africa	Enablers: Integration of HIV care in PHC – patient satisfaction and quality of care	Level III B
				Barriers: Possible knock on effect on other services (child health lacking, scores higher for TB attendees compared with chronic disease care attendees)	
Sekatane^41^ *(Master’s thesis)*	To develop protocol for professional nurses regarding NIMART management that is based on data and specific challenges that are faced in the Ehlanzeni district by professional nurses	Quantitative: Cross-sectional questionnaire	A total of 135 professional nurses who are NIMART trained	Barriers: Lack of professional nurses, fear of infecting themselves whilst treating patients, shortage of ART, lack of doctor support, patients not coming for treatment, not able to trace defaulters	Level III B
Solomons et al.^22^	To investigate factors influencing the knowledge and confidence of professional nurses in managing patients living with HIV in PHC settings in a rural and urban district in the Western Cape	Quantitative: A cross-sectional survey	A total of 77 NIMART-trained nurses from 29 healthcare facilities	Enablers: Training on guidelines, knowledge and confidence, support and regular feedback about personal performance, adequate 2 weeks of mentoring, caseload – frequently managing persons living with HIV in practice	Level III B
Swart et al.^42^	To describe the queries received from nurses by the hotline between 01 March and 31 May 2012 and identify problem areas and knowledge gaps where nurses may require further training	Quantitative: Retrospective record review	A total of 1479 HIV- and TB-related queries from healthcare workers	Barriers: Not all nurses NIMART trained, knowledge gaps of nurses – interpretation of laboratory results before initiating ART	Level III B
Visser et al.^32^	To evaluate the quality of care provided, the barriers to the effective rollout of antiretroviral services and the role of a clinical mentor	Mixed methods: Data were collected using patient satisfaction surveys, review of clinical records, facility audits, focus group interviews, field notes and a reflection diary	A total of 537 clinical records A total of 354 patient satisfaction surveys focus groups participants (*n* = 15)	Enablers: Ongoing mentoring and support, following guidelines, rational prescription, patient satisfaction	Level III B
				Barriers: Salary challenges, excessive workload, lack of trained nurses, infrastructural barriers, drug shortages	
Williams et al.^29^	To explore the experiences of healthcare professionals regarding the provision of ART for children at PHC clinics	Qualitative: In-depth interviews	A total of 19 interviews with healthcare professionals	Barriers: Lack of resources, need for training and mentoring and debriefing, disharmony in the work environment, ineffective management, non-conducive work environment, incongruence in the interpretation of side-effects of ART in children, apprehension to work with children, lack of patient attendance and adherence	Level III B
Xaba^28^ *(Master’s thesis)*	To conduct an implementation evaluation study of the NIMART programme in PHC clinics in the Ugu district of KwaZulu-Natal	Quantitative: Cross-sectional questionnaires	A total of 52 professional nurses	Enablers: Nurses initiating patients in practice (lower for children); nurses’ knowledge on ART regimens, eligibility and monitoring good on average, some knowledge gaps identified, availability of latest guidelines	Level III B
Zuber et al.^46^	To describe the extent of NIMART in practice, education, policy and regulation in East, Central and Southern Africa	Quantitative: Survey	Senior nursing leadership teams from 15 African countries	Barriers: NIMART authorised in policy but not reinforced by regulation nor incorporated into pre-service education	Level III B
*Key for levels of evidence:*^13^ *Level I: Experimental, RCT, systematic review or RCTs* *Level II: Quasi-experimental study, systematic review of quasi-experimental/combined with RCTs* *Level III: Non-experimental, qualitative studies, systematic reviews of non-experimental studies* *Level IV: Opinions, clinical practice guidelines, consensus panels* *A: High quality* *B: Good quality* *C: Poor quality, major flaw*					

HIV, human immunodeficiency virus; NIMART, nurse-initiated and managed antiretroviral treatment; PHC, primary healthcare; ART, antiretroviral treatment; STRETCH, Streamlining Tasks and Roles to Expand Treatment and Care for HIV.

## References

[1] Simelela NP, Venter WDF. A brief history of South Africa’s response to AIDS. S Afr Med J. 2014;104(3):249–251. 10.7196/SAMJ.7700 24893502

[2] Mngqibisa R, Muzigaba M, Ncama BP, Pillay S, Nadesan-Reddy N. Upskilling nursing students and nurse practitioners to initiate and manage patients on ART: An outcome evaluation of the UKZN NIMART course. African J Heal Prof Educ. 2017;9(3):153. 10.7196/AJHPE.2017.v9i3.879

[3] Kredo T, Bateganya M, Pienaar ED, Adeniyi FB. Task shifting from doctors to non-doctors for initiation and maintenance of HIV/AIDS treatment. Cochrane Database Syst Rev. 2014;(7):CD007331. 10.1002/14651858.CD007331.pub2 24980859 PMC11214583

[4] Emdin CA, Chong NJ, Millson PE. Non-physician clinician provided HIV treatment results in equivalent outcomes as physician-provided care: A meta-analysis. J Int AIDS Soc. 2013;16:18445. 10.7448/IAS.16.1.18445 23827470 PMC3702014

[5] Kredo T, Ford N, Adeniyi FB, Garner P. Decentralising HIV treatment delivery in middle-and low-income countries. Cochrane Database Syst Rev. 2013;(7):CD009987. 10.1002/14651858.CD009987 23807693 PMC10009870

[6] World Health Organization. Task shifting: Global recommendations and guidelines [homepage on the Internet]. Vol. 53. UNAIDS; 2008 [cited 2020 Oct 07]. Available from: https://www.who.int/workforcealliance/knowledge/resources/taskshifting_guidelines/en/

[7] Colvin CJ, Fairall L, Lewin S, et al. Expanding access to ART in South Africa: The role of nurse-initiated treatment. SAMJ. 2010;100(4):210–212. 10.7196/SAMJ.4124 20459957

[8] Mathibe MD, Hendricks SJH, Bergh AM. Clinician perceptions and patient experiences of antiretroviral treatment integration in primary health care clinics, Tshwane, South Africa. Curationis. 2015;38(1):1–11. 10.4102/curationis.v38i1.1489 PMC6091789 26841916

[9] Makhado L, Davhana-Maselesele M, Farley JE. Barriers to tuberculosis and human immunodeficiency virus treatment guidelines adherence among nurses initiating and managing anti-retroviral therapy in KwaZulu-Natal and North West provinces. Curationis. 2018;41(1):1–8. 10.4102/curationis.v41i1.1808 PMC6091579 29781696

[10] Mnyani CN, Marinda E, Struthers H, Gulley M, Machepa R, McIntyre J. Timing of antenatal care and ART initiation in HIV-infected pregnant women before and after introduction of NIMART. S Afr J HIV Med. 2014;15(2):55–56. 10.7196/SAJHIVMED.1009

[11] Republic of South Africa. National Department of Health. ART clinical guidelines for the management of HIV in adults, pregnancy, adolescents, children, infants and neonates [homepage on the Internet]. 2019 [cited 2020 Oct 07]. Available from: https://www.knowledgehub.org.za/elibrary/2019-art-clinical-guidelines-management-hiv-adults-pregnancy-adolescents-children-infants

[12] Green BN, Johnson CD, Adams A. Writing narrative literature reviews for peer-reviewed journals: Secrets of the trade. J Chiropr Med. 2006;5(3):101–117. 10.1016/S0899-3467(07)60142-6 19674681 PMC2647067

[13] Johns Hopkins. Johns Hopkins nursing evidence-based practice appendix C: Evidence level and quality guide evidence levels: 1–3 [homepage on the Internet]. [cited 2020 Oct 07]. Available from: https://www.hopkinsmedicine.org/evidence-based-practice/_docs/appendix_c_evidence_level_quality_guide.pdf

[14] Georgeu D, Colvin CJ, Lewin S, et al. Implementing nurse-initiated and managed antiretroviral treatment (NIMART) in South Africa: A qualitative process evaluation of the STRETCH trial. Implement Sci. 2012;7(1):1–13. 10.1186/1748-5908-7-66 PMC3464669 22800379

[15] Jones M, Cameron D. Evaluating 5 years’ NIMART mentoring in South Africa’s HIV treatment programme: Successes, challenges and future needs. South African Med J. 2017;107(10):839–842. 10.7196/SAMJ.2017.v107i10.12392 29022525

[16] South African Department of Health. Clinical mentorship manual for integrated services [homepage on the Internet]. 2011 [2020 Oct 07]. Available from: https://sahivsoc.org/Files/clinical%20mentorship%202011.pdf

[17] Green A, De Azevedo V, Patten G, Davies MA, Ibeto M, Cox V. Clinical mentorship of nurse initiated antiretroviral therapy in Khayelitsha, South Africa: A quality of care assessment. PLoS One. 2014;9(6):e98389. 10.1371/journal.pone.0098389 24887260 PMC4041881

[18] Crowley T. The effect of a HIV/TB competency-based short course on professional nurses’ knowledge and confidence in managing patients with HIV and TB. In: Annual Nursing Education Conference (ANEC), South Africa. 2013. [conference proceedings not published]

[19] Cameron D, Gerber A, Mbatha M, Mutyabule J, Swart H, West N. Nurse initiation and maintenance of patients on antiretroviral therapy: Are nurses in primary care clinics initiating ART after attending NIMART training. SAMJ. 2012;102(2):98–100. 10.7196/SAMJ.5195 22310442

[20] Davies NECG, Homfray M, Venables EC. Nurse and manager perceptions of nurse initiated and managed antiretroviral therapy (NIMART) implementation in South Africa: A qualitative study. BMJ Open. 2013;3(11):e003840. 10.1136/bmjopen-2013-003840 PMC3831110 24240142

[21] Naude SM. Framework to empower professional nurses for nurse initiated management of anti-retroviral therapy North West province. Sefako Makgatho Health Sciences University; 2017 [cited 2020 Apr 8]. Available from: https://repository.smu.ac.za/xmlui/bitstream/handle/20.500.12308/463/S%20Naude%20thesis%20final%20February%202018%20.pdf?sequence=1&isAllowed=y

[22] Solomons DJ, Van der Merwe AS, Esterhuizen TM, Crowley T. Factors influencing the confidence and knowledge of nurses prescribing antiretroviral treatment in a rural and urban district in the Western Cape province. South Afr J HIV Med. 2019;20(1):1–7. 10.4102/sajhivmed.v20i1.923 PMC6620519 31308969

[23] Mabelane T, Marincowitz GJO, Ogunbanjo GA, Govender I. Factors affecting the implementation of nurse-initiated antiretroviral treatment in primary health care clinics of Limpopo Province, South Africa. S Afr Fam Pract. 2016;58(1):9–12. 10.1080/20786190.2015.1114704

[24] Motlokoa MN. Factors influencing submission of portfolios of evidence amongst nurses trained in nurse initiation and management of antiretroviral therapy in North West. University of the Witwatersrand; 2016 [cited 2020 Apr 8]. Available from: http://wiredspace.wits.ac.za/bitstream/handle/10539/22461/Final%20Research%20Report%20-(2016-10-21)edited.pdf?isAllowed=y&sequence=1

[25] Rasalanavho RN. Nurses implementing the nurse-initiated- and-managed antiretroviral treatment programme in Vhembe district, South Africa. University of Venda; 2015 [cited 2020 Apr 7]. Available from: https://www.semanticscholar.org/paper/Challenges-confronting-professional-nurses-the-and-Rasalanavho/c0fa7dea657ec59795bede7ab214f25159161803

[26] Nyasulu JCY, Muchiri E, Mazwi S, Ratshefola M. NIMART rollout to primary healthcare facilities increases access to antiretrovirals in Johannesburg: An interrupted time series analysis. SAMJ. 2013;103(4):232–236. 10.7196/SAMJ.6380 23547688

[27] Mophosho Z. The implementation of nurse initiated and managed antiretroviral therapy in the City of Johannesburg clinics: Perceived facilitators and barriers. University of Witwatersrand; 2015 [cited 2020 Apr 7]. Available from: http://wiredspace.wits.ac.za/handle/10539/18539

[28] Xaba P. An implementation evaluation study of a nurse initiated and managed antiretroviral therapy (NIMART) program in primary health care clinics in the Ugu district of kKwazulu-Natal. University of KwaZulu-Natal; 2016 [cited 2020 Apr 7]. Available from: https://researchspace.ukzn.ac.za/bitstream/handle/10413/15808/Xaba_Pearl_2016.pdf?sequence=1&isAllowed=y

[29] Williams M, Van Rooyen DRM, Ricks EJ. Provision of antiretroviral therapy for children in Nelson Mandela Bay: Health care professionals’ challenges. African J Prim Heal Care Fam Med. 2018;10(1):1–11. 10.4102/phcfm.v10i1.1490 PMC5913768 29781680

[30] Jones M, Stander M, Van Zyl M, Cameron D. Recall of lost-to-follow-up pre-antiretroviral therapy patients in the Eastern Cape: Effect of mentoring on patient care. SAMJ. 2012;102(9):768–769. 10.7196/SAMJ.5957 22958703

[31] Jobson G, Mabitsi M, Railton J, et al. Targeted mentoring for human immunodeficiency virus programme support in South Africa. South Afr J HIV Med. 2019;20(1):1–6. 10.4102/sajhivmed.v20i1.873 PMC6407314 30863623

[32] Visser CA, Wolvaardt JE, Cameron D, Marincowitz GJO. Clinical mentoring to improve quality of care provided at three NIM-ART facilities: A mixed methods study. African J Prim Heal Care Fam Med. 2018;10(1):1–7. 10.4102/phcfm.v10i1.1579 PMC6018522 29943605

[33] Makhado L, Davhana-Maselesele M, Lebese R, Maputle S. Factors facilitating trained NIMART nurses ’ adherence to treatment guidelines: A vital matter in the management of TB/HIV treatment in South Africa. BMC Nursing. 2020;19:77. 10.1186/s12912-020-00470-6 32821244 PMC7429774

[34] Nozulu N, Gaede BM. Antiretroviral initiation of pregnant women and antenatal care booking practices in eThekwini District, KwaZulu-Natal, South Africa. Afr J Prm Health Care Fam Med. 2018;10(1):a1606. 10.4102/phcfm.v10i1.160 PMC6018727 29943591

[35] Mashudu M. An evaluation of the effectiveness of the nurse-initiated-and-managed antiretroviral treatment (NIMART) programme, Waterberg District, Limpopo Province. University of Venda; 2015.

[36] Mangi NG, Goon D Ter, Yako EM. Self-efficacy and clinical performance of nurses initiated and management of antiretroviral therapy: Narrative review. Open Publ Health J. 2019;12:86–93. 10.2174/1874944501912010086

[37] Mboweni SH, Makhado L. Conceptual framework for strengthening nurse-initiated management of antiretroviral therapy training and implementation in North West Province. Healh SA Gesondheid. 2020;25:1–10. 10.4102/hsag.v25i0.1285 PMC7059635 32161674

[38] Stellenbosch University. ART2Scale final technical report update 25 Nov. 2010.

[39] Mboweni SH, Makhado L. Challenges influencing nurse-initiated management of antiretroviral therapy training and implementation in Ngaka Modiri Molema district, North West province. Heal SA Gesondheid. 2020;25:1–11. 10.4102/hsag.v25i0.1174 PMC7136686 32284885

[40] Mboweni SH, Makhado L. Impact of NIMART training on HIV management in Ngaka Modiri Molema District, North West province. Int J Africa Nurs Sci. 2019;11(January):100170. 10.1016/j.ijans.2019.100170

[41] Sekatane PT. A protocol for professional nurses regarding the management of nurse initiated management of antiretroviral therapy (NIMART) in the Ehlanzeni district, Mpumalanga province, South Africa. University of Limpopo; 2014 [cited 2020 Apr 7]. Available from: http://ulspace.ul.ac.za/handle/10386/1377

[42] Swart AM, Chisholm BS, Cohen K, Workman LJ, Cameron J, Blockman M. Analysis of queries from nurses to the South African National HIV & TB health care worker hotline. South Afr J HIV Med. 2013;14(4):179–182. 10.7196/SAJHIVMED.948

[43] Hanrahan BA, Williams A. Prevention of mother-to-child transmission of HIV guidelines: Nurses’ views at four primary healthcare facilities in the Limpopo Province. S Afr J HIV Med. 2017;18(1):1–6. 10.4102/sajhivmed.v18i1.690 PMC5843237 29568626

[44] Rawat A, Uebel K, Moore D. Patient responses on quality of care and satisfaction with staff after integrated HIV care in South African primary health care clinics. J Assoc Nurses AIDS Care. 2018;29(5):698–711. 10.1016/j.jana.2018.04.014 29857926

[45] Ford P. Nurse initiated and managed anti-retroviral treatment: An ethical and legal analysis in South Africa. MSc Med (Bioethics Health Law). Johannesberg; 2013 [cited 2020 Apr 7]. Available from: http://wiredspace.wits.ac.za/bitstream/handle/10539/14413/Research_Report_NIMART__Final_Submission_with_Amendments-20-07-13.pdf?sequence=1

[46] Zuber A, McCarthy CF, Verani AR, Msidi E, Johnson C. A survey of Nurse-Initiated and -Managed Antiretroviral Therapy (NIMART) in practice, education, policy, and regulation in East, Central, and Southern Africa. J Assoc Nurses AIDS Care. 2014;25(6):520–531. 10.1016/j.jana.2014.02.003 24739661

[47] Lekhuleni ME, Kgole JC, Mbombi MO. Knowledge of student nurses in nurse initiated and management of antiretroviral therapy at the University of Limpopo, South Africa: TB, HIV/AIDS and other diseases. African J Phys Heal Educ Recreat Danc. 2015;21(October):53–61.

[48] Modeste RM, Adejumo O. Strengthening the preparation of student nurses to participate in the provision of nurse initiated and monitored Antiretroviral (NIMART) in South Africa [homepage on the Internet]. 2016 [cited 2020 Oct 07]. Available from: https://hdl.handle.net/10755/603007

[49] Colvin CJ, Fairall L, Lewin S, et al. Expanding access to ART in South Africa: The role of nurse-initiated treatment. SAMJ. 2010;100(4):210–211. 20459957 10.7196/samj.4124

